# Clinical, cytogenetic, and molecular findings in a fetus with ultrasonic multiple malformations, 4q duplication, and 7q deletion: A case report and literature review

**DOI:** 10.1097/MD.0000000000013094

**Published:** 2018-11-09

**Authors:** Fagui Yue, Yuting Jiang, Yang Yu, Xiao Yang, Hongguo Zhang, Ruizhi Liu, Ruixue Wang

**Affiliations:** a Center for Reproductive Medicine and Center for Prenatal Diagnosis, First Hospital; b Jilin Engineering Research Center for Reproductive Medicine and Genetics, Jilin University, Changchun, China.

**Keywords:** 4q duplication, 7q deletion, abnormal ultrasound, de novo, FISH, SNP array

## Abstract

**Rationale:**

Chromosome deletion/duplication has been reported to be associated with mental disability and dysmorphism according to the accumulated research evidence.

**Patient concerns:**

A 25-year-old woman underwent amniocentesis for cytogenetic and single-nucleotide polymorphism (SNP) array analysis at 18 weeks of gestation due to the increased Down syndrome risk of 1/13.

**Diagnoses:**

The fetal chromosomal analysis revealed a seemingly “normal” chromosomal karyotype, but the SNP array results showed a partial duplication of chromosome 4q34.1q35.2 and a deletion of chromosome 7q34q36.3fluorescence in situ hybridization (FISH) analysis showed that the couple had normal chromosome 4 and 7, whereas there was a partial signal fragment of chromosome 4 attached on the long arm of chromosome 7 for the fetus.

**Interventions:**

The couple finally chose to terminate the pregnancy based on the ultrasonic multiple malformations and the abnormal SNP array results.

**Outcomes:**

The duplicated/deleted segments of the fetus were de novo. Meanwhile, we consider *SHH* and *XRCC2* as good candidate genes, which may, in part, explain the observed abnormalities for the fetus.

**Lessons:**

The combination of SNP array and FISH analysis can give a molecular chromosomal diagnosis, which will offer more clear cytogenetic diagnosis and genetic counseling.

## 1 Introduction

Trisomy 4q syndrome was first described in 1970s, which has been already described in more than 60 patients. The main clinical features include growth and psychomotor retardation, microcephaly, large low-set ears, prominent nasal bridge, and so on.^[1,2]^ In fact, “pure” 4q duplication reports are uncommon, the spanning region of which is usually from q12 to q35. Meanwhile, most of the 4q duplications have been derived from a parental balanced translocation, associated with various 4q trisomy and partial monosomy of other chromosomes. The variable clinical phenotypes observed in these patients are mainly related to the 4q duplicated segments, and also the corresponding monosomy, which cause the difficult genotype–phenotype correlation.^[3,4]^ Although the pure duplications are less common, they serve as a basis for a better understanding of relationships between chromosomal anomalies and their clinical manifestations.^[3]^ Also, it is widely accepted that distal 4q duplications, rather than proximal ones, seem to cause a more severe clinical syndrome.^[5]^

Terminal deletion of 7q was first described in 1960s, which is a rare chromosomal structural loss associated with long arm of chromosome 7. Patients with 7q terminal deletions often present growth and motor retardation, intellectual disability, hypotelorism, hypotonia, microcephaly, upslanting palpebral fissures, prominent forehead, epicanthal folds, cleft lip, flat and broad nasal bridge, bulbous nasal tip, micrognathia, abnormal palmar crease, feeding problems, and low set ears.^[6]^ The frequencies of chromosome 7q terminal deletions are higher compared with interstitial deletions based on the published literature, and the 7q terminal deletion often show severe phenotypic features including sacral agenesis and holoprosencephaly (HPE).^[7]^ Rush et al^[8]^ speculated that distal interstitial deletions of chromosome 7q might represent a recognizable phenotype and could be considered a separate deletion syndrome.

Here, we report a fetus with a de novo duplication of chromosome 4q34.1q35.2 and a deletion of chromosome 7q34q36.3, consisting of abnormal sonography findings. Meanwhile, we also compare the similarities of the clinical features of the cases consisting of 4q duplication and 7q deletion as described in the literature.

## 2 Case report

**Figure 1:**
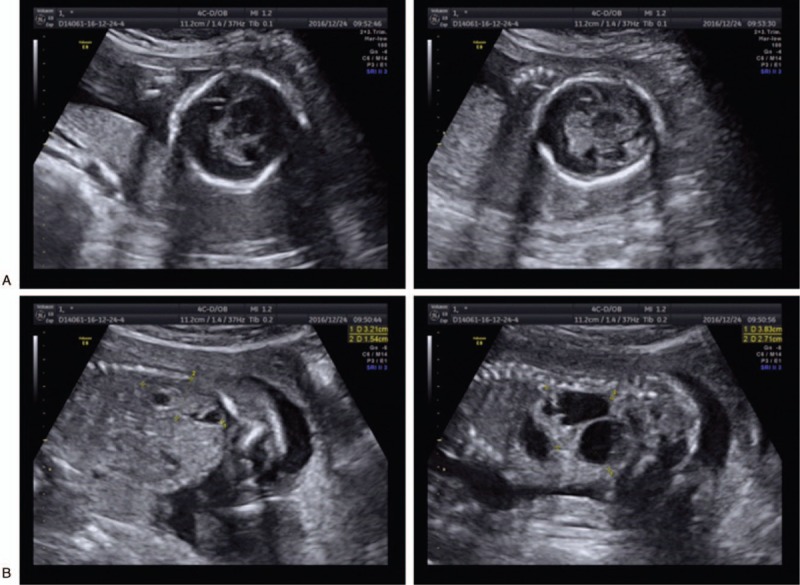
Prenatal ultrasound at 20 weeks of gestation shows the fetus with single ventricle in intracalvarium, thalmus partially fused (A) and multicystic dysplastic kidneys (B).

A 25-year-old, gravida 1, para 0, woman underwent amniocentesis for cytogenetic and single-nucleotide polymorphism (SNP) array analysis at 18 weeks of gestation because of the increased Down syndrome risk of 1/13, calculated from a low maternal serum alpha fetoprotein (AFP) level of 0.820 multiple of median (MoM), a low uE3 level of 0.178 MoM, and a high human chorionic gonadotropin (hCG) level of 4.574 MoM in the second trimester. Meanwhile, 20 weeks’ sonography findings indicated the abnormalities of single ventricle in intracalvarium, thalmus partially fused, and polycystic kidneys (Fig. 1). She and her husband were nonconsanguineous and healthy. There was no family history of diabetes mellitus or congenital malformations. The mother denied any exposure to alcohol, teratogenic agents, irradiation, or infectious diseases during this pregnancy. The study protocol was approved by the Ethics Committee of the First Hospital of Jilin University, and written informed consent was obtained from the couple.

## 3 Methods

### 3.1 Cytogenetic analysis

Chromosome analysis was performed on G-band metaphases prepared from the cultured amino fluid cells according to the laboratory's standard protocols. Twenty metaphases were analyzed for this sample. The International System for Human Cytogenetic Nomenclature (ISCN 2013) nomenclature was used to describe the karyotype.^[9]^ Then the couple were obtained for karyotyping because of the abnormal SNP array results of the fetus after obtaining written informed consent.

### 3.2 SNP array analysis

The SNP array analysis was performed using Human CytoSNP-12 BeadChip (Illumina, San Diego, CA). The DNA was extracted from 10 mL of uncultured amino fluid cells using Qiagen micro kit. The data of these images were analyzed according to Illumina's Genome Studio software. The final results were analyzed through the Database of Chromosomal Imbalance and Phenotype in Humans using Ensemble Resources (DECIPHER), database of genomic variants (DGV), Online Mendelian Inheritance in Man, National Center for Biotechnology Information, and so on.^[10]^

### 3.3 Fluorescence in situ hybridization analysis

Following the results of cytogenetic analysis and SNP array results, fluorescence in situ hybridization (FISH) using whole chromosome painting probes specific for chromosome 4 and 7 (Cytocell Technologies, Cambridge, UK) were performed on metaphase slides for the fetus and the couple according to the manufacturer's protocol.

## 4 Results

**Figure 2:**
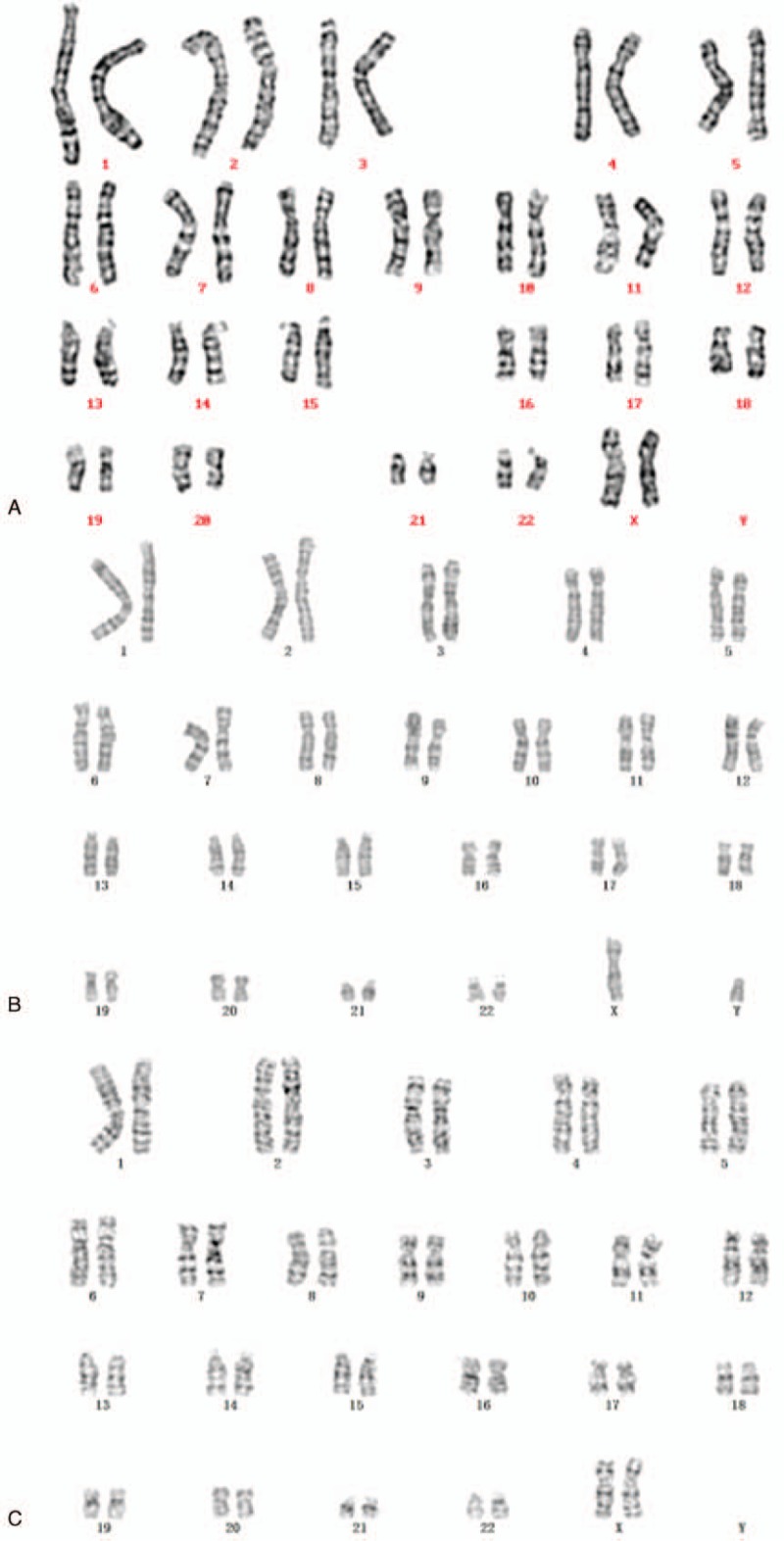
(A) Karyotype of the fetus identified by GTG banding technique. (B) The father's karyotype. (C) The mother's karyotype.

**Figure 3:**
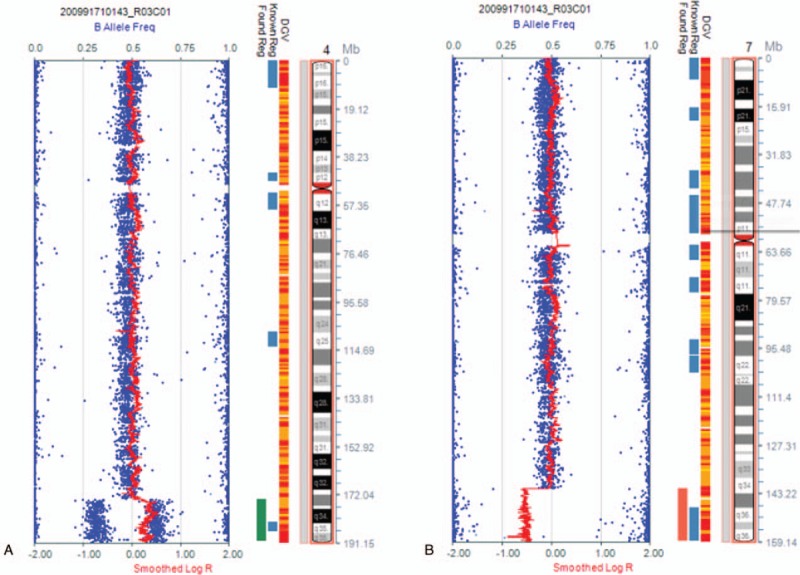
Single-nucleotide polymorphism (SNP) array on uncultured amniocytes depicted 4q34.1q35.2 duplication (A) and 7q34q36.3 deletion (B).

**Figure 4:**
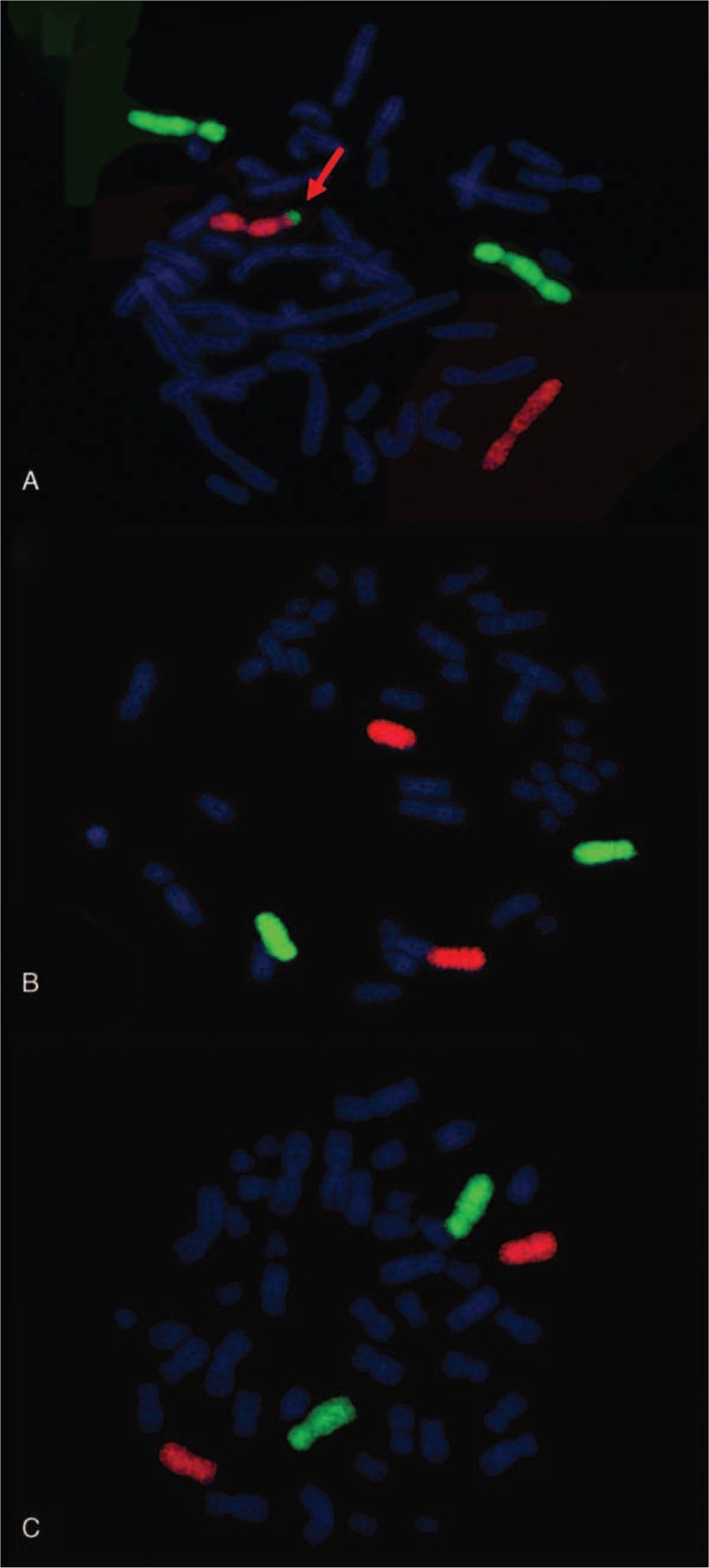
Metaphase-FISH results of whole chromosome 4 (green) and 7 (red) painting probe. (A) The fetus. Arrow indicates partially duplicated chromosome 4 attached on the long arm of chromosome 7. (B) The father's. (C) The mother's.

The fetus was initially found to have a seemingly normal karyotype of 46,XX by routine cytogenetic analysis (Fig. 2A). However, SNP array revealed a 17-Mb duplication of 4q34.1q35.2 and a 17-Mb deletion of 7q34q36.3 (4q34.1q35.2 [173,822,571–190,880,409] × 3; 7q34q36.3 [144,672,604–159,119,486] × 1) (Fig. 3). The abnormal SNP array results may cater for the sonography abnormalities to some extent. Afterwards, we informed the parents to perform peripheral chromosome studies, and conventional cytogenetic analysis demonstrated the father (Fig. 2B) and the mother (Fig. 2C) both had normal chromosomal karyotypes. To further define the chromosome abnormalities were de novo or inherited from parental unbalanced translocation, the whole chromosome painting probes specific for chromosome 4 and 7 were applied subsequently. The chromosome 4 painting probe was found to hybridize to the terminal of chromosome 7q on the fetus, thus confirming the existence of a partial 4q duplication (Fig. 4A). Also, the father (Fig. 4B) and the mother (Fig. 4C) showed normal whole painting chromosome 4 and 7. So we finally got the conclusion that the chromosome aberrations of the fetus detected through SNP array were de novo. The couple finally chose to terminate the pregnancy based on the abnormal ultrasonography and SNP array results.

## 5 Discussion

We describe a fetus with single ventricle in intracalvarium, thalmus partially fused, and polycystic kidneys, carrying de novo 4q34.1q35.2 duplication and 7q34q36.3 deletion identified by SNP array and FISH analysis. Duplications of 4q represent rare chromosomal abnormalities, which usually result from an unbalanced segregation of a familial translocation and lead to a variety of congenital anomalies.^[11]^ The common clinical features of pure 4q duplication are summarized to further investigate the genotype–phenotype correlation in Table 1.^[1,3,11–17]^

**Table 1:**
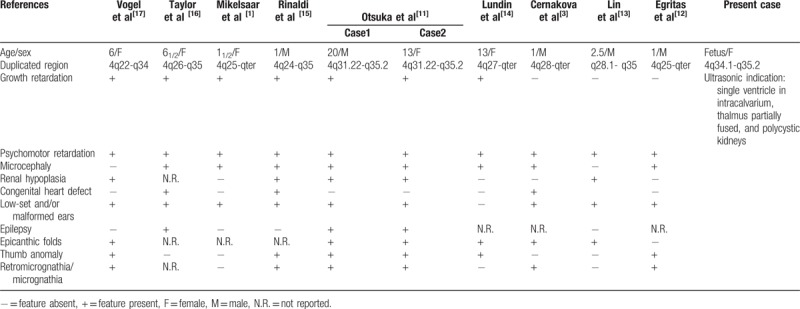
Summary of clinical manifestations in patients with duplication of overlapping segment 4q34.1-q35.2.

The most frequent breakpoints of 4q duplication occurs between 4q21 and 4q28.^[2]^ Rinaldi et al^[15]^ described a newborn of 4q24qter duplication with severe psychomotor retardation, minor anomalies, congenital heart defects, and thumb and urogenital abnormalities. Mikelsaar et al^[1]^ reported a partial trisomy 4q25-qter case with clinical features to be growth retardation, psychomotor retardation, microcephaly, large, low set, malformed ears, prominent nasal bridge, ptosis, and epicanthus. The fragment 4q27-4q31 might be relevant to severe clinical effects, including growth retardation, mental retardation, microcephaly, facial asymmetry, thumb anomalies, hearing impairment, epilepsy, and congenital heart defect.^[18]^ Cernakova et al^[3]^ introduced a newborn of dup(4)(q28q35.2) with hypotrophy and somatic stigmatization: microcephaly, facial dysmorphism, heart defect, and immunodeficiency syndrome. The region 4q31-q33 might be involved in the development of the 4q characteristic dysmorphic characteristic.^[4]^ Thapa et al^[2]^ deduced that the duplication of 4q32-qter might be associated with developmental delay/mild-to-moderate intellectual disability, cranial abnormalities, minor anomalies of the face, and digits. However, Kim et al^[19]^ delineated a male patient showing an almost normal phenotype except for congenital dysfunction in spermatogenesis, with pure trisomy 4q32-35. Elghezal et al^[4]^ proposed that the region 4q35 might be involved in the development of microcephaly, and severe mental and growth retardation. To our knowledge, our case has a relatively small trisomic region 4q34.1q35.2, which was first reported and different from the reviewed literature before. As more and more research on molecular characterization of 4q duplication become available, the genotype–phenotype correlation may become clearer.

There have been relevant literature reporting the cases of 7q interstitial and terminal deletion according to the first/second maternal serum screening.^[20–22]^ In our report, the fetus presented a 7q34q36.3 deletion, together with positive screen risk of 1/13 for trisomy 21 and abnormal sonography findings. But additional supported research is needed to verify whether there is an actual correlation between 7q deletion and abnormal maternal serum screening.

The main clinical findings of 7q deletion syndrome include growth restriction, developmental retardation, microcephaly, HPE, ocular abnormalities, a flat and broad nasal bridge, genital anomalies, a prominent forehead, ocular hypertelorism, cleft lip and palate, micrognathia, large low set malformed ears, abnormal fingers, and so on.^[21,23]^ We compare the similar deletion regions and clinical features in patients with 7q34-q36.3 overlapped in Table 2,^[6,8,23–29]^ which present common characteristics as shown. Roessler et al^[28]^ and Jackson et al^[26]^ separately reported deletions of 7q34-qter and 7q34-q36.3; the sharing clinical phenotype is microcephaly.

**Table 2:**
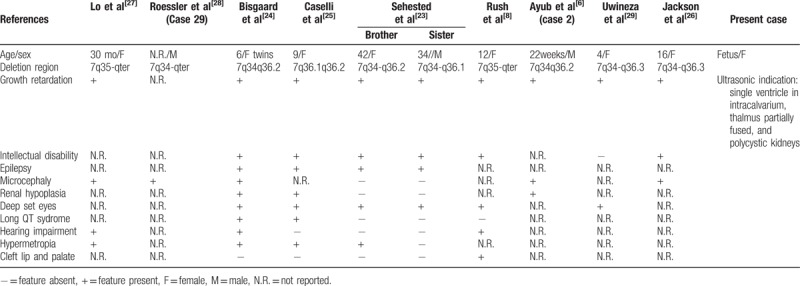
Summary of clinical manifestations in patients with deletion of overlapping segment 7q34-q36.3.

**Figure 5:**
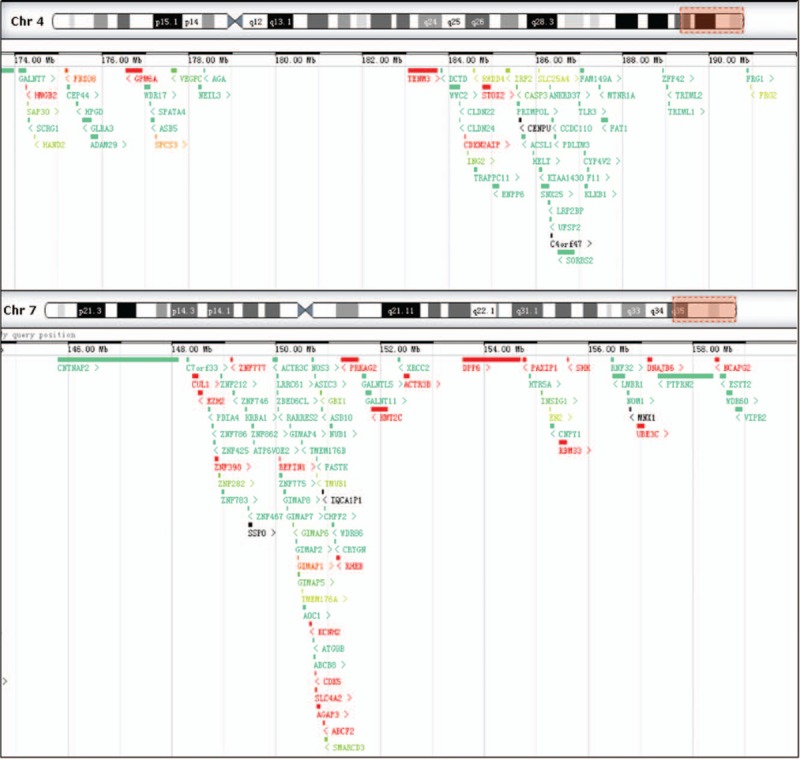
The involving genes contained in the region of 4q34.1-4q35.2(4:173,822,571–190,880,409) and 7q34-7q36.3(7:144,672,604–159,119,486) seen in our case. The figure is modified from DECIPHER genome browser.

The SNP array analysis revealed that the fetus had trisomy 4q34.1-4q35.2 and monosomy 7q34-7q36.3. All the involving genes in 4q34.1-4q35.2 (4:173,822,571-190,880,409) and 7q34-7q36.3 (7:144,672,604-159,119,486) are shown in Fig. 5. Meanwhile, these 2 regions above each contain more than 10 morbid genes including *HPGD,VEGFC, AGA, TENM3, TRAPPC11, PRIMPOL, SLC25A4, CYP4V2, TLR3, KLKB1*, and *F11* in the region of 4q34.1-4q35.2; and *CNTNAP2, EZH2, KCNH2, NOS3, CDK5, ASB10, PRKAG2, KMT2C, XRCC2, DPP6, SHH, LMBR1, DNAJB6, MNX1,* and *WDR60* in the region of 7q34-7q36.3. These genes and the corresponding diseases have been summarized in Table 3. Based on the published literature and ultrasonic clinical manifestations, we consider *SHH* and *XRCC2* as good candidate genes, which may, in part, explain the observed abnormalities for the fetus.

**Table 3:**
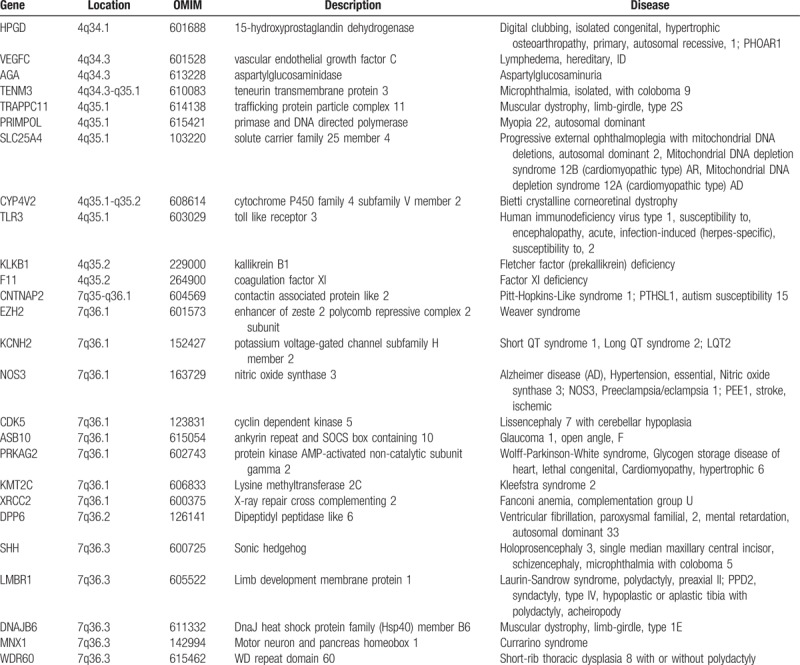
Genes in the region of 4q34.1-4q35.2 and 7q34-7q36.3 and the associated diseases.

The *SHH* gene, located in 7q36.3, encodes sonic hedgehog, which is a secreted protein involved in the developments of embryo, the brain and spinal cord, eyes, limbs abnormalities, and so on. Haploinsufficiency of *SHH* gene was associated with HPE, and the mutations of *SHH* gene were the most common cause of nonsyndromic HPE.^[30,31]^ It has been widely accepted that the dysfunction of hedgehog signaling is a common mechanism for the production of HPE-like phenotypes. The abnormal ultrasonic findings indicated that our case presented with HPE, which is consistent with previous reports.

Renal ultrasound abnormalities are described in our case. Otsuka et al^[11]^ suggested that renal hypoplasia might be female-prone and probably have a close relationship with duplication of 4q33-q34. Our case happened to be a female with a duplication of 4q34.1q35.2 and abnormal kidney ultrasound findings, which aroused our interest that 4q34 may be an interesting region in renal hypoplasia. In addition, Jackson et al^[26]^ speculated that renal malformations may mainly be associated with 7q36.1-qter deletion and 7q35 deletion, and there maybe exist genitourinary development putative genes at these regions. Also, the research of Caselli et al^[25]^ suggested that the deletion of 7q36.1 and 7q36.2 have more links with renal hypoplasia. The *XRCC2* gene localized on 7q36.1 is a member of the RAD51 gene family, which encodes proteins involved in homologous recombination repair of DNA damage.^[32]^ Malformed kidneys were reported in the associated diseases of *XRCC2* gene.^[33]^

It is hard to detect the chromosome rearrangements with similar size and banding patterns by routine karyotype. However, the application of SNP array can describe the breakpoints and involved functional genes more precisely.^[34]^ In our case, the combination of SNP array and FISH analysis gives a molecular chromosomal diagnosis, demonstrating that the 4q duplication and 7q deletion are de novo, not inherited. So, further prenatal consultation for the couple can be given appropriately.

## 6 Conclusions

In conclusion, we reported a fetus with de novo trisomy 4q34.1q35.2 and monosomy 7q34q36.3, showing abnormal maternal serum screening and sonography findings. To our knowledge, this is the first report of prenatally diagnosed 4q duplication and 7q deletion by a second-trimester screening for Down syndrome. Moreover, the duplication/deletion regions in our report are not pure, so we cannot make a conclusion that the ultrasonic abnormalities are associated with 4q duplication and/or 7q deletion. In this case, molecular genetic techniques help us in the precise detection and verification as important tools, which prove that accurate characterization of abnormal chromosomes is important for genetic counseling in clinic. Meanwhile, we consider *SHH* and *XRCC2* as good candidate genes which may in part explain the HPE and renal abnormalities for the fetus.

## Author contributions

**Conceptualization:** Ruixue Wang.

**Data curation:** Hongguo Zhang, Ruixue Wang.

**Formal analysis:** Hongguo Zhang.

**Funding acquisition:** Ruizhi Liu.

**Investigation:** Yang Yu.

**Methodology:** Yuting Jiang.

**Project administration:** Ruizhi Liu.

**Software:** Xiao Yang.

**Validation:** Ruizhi Liu.

**Visualization:** Ruizhi Liu, Ruixue Wang.

**Writing – original draft:** Fagui Yue.

**Writing – review & editing:** Ruixue Wang.
